# Targeting Cancer Cachexia: A Mechanistic Evaluation of Anti‐GDF‐15 Antibody‐Based Combination Therapies

**DOI:** 10.1002/jcsm.70312

**Published:** 2026-05-13

**Authors:** Danna M. Breen, Stephanie Joaquim, Brianna LaCarubba Paulhus, Donald Bennett, Barbara Bernardo, Susie Collins, Ryan M. Esquejo, Ja Young Kim‐Muller, Laura Lin, Matthew Peloquin, Zhidan Wu, Shuxi Qiao, John C. Stansfield, Bei B. Zhang, Michelle I. Rossulek

**Affiliations:** ^1^ Internal Medicine Research Unit, Pfizer Inc Cambridge Massachusetts USA; ^2^ Biostatistics, Early Clinical Development, Pfizer Inc Cambridge Massachusetts USA; ^3^ Statistics, Internal Medicine Research Unit, Pfizer R&D UK Limited Cambridge UK; ^4^ Biomedicine Design, Pfizer Inc Cambridge Massachusetts USA

**Keywords:** cachexia, GDF‐15, ghrelin, mouse tumour models, myostatin

## Abstract

**Background:**

In a recent Phase 2 trial in patients with cancer cachexia, the anti‐GDF‐15 antibody ponsegromab resulted in increased body weight, appetite, muscle mass and physical activity. This study provides compelling evidence that targeting the GDF‐15 pathway may offer a viable therapeutic strategy, while raising new mechanistic questions about how GDF‐15 neutralization could be optimally integrated with other interventions to reverse the multifactorial cachexia syndrome. This series of experiments aimed to evaluate the effects of anti‐GDF‐15 antibody treatment in combination with muscle anabolic (anti‐myostatin antibody) or appetite stimulant (ghrelin receptor agonist anamorelin) modulators using mouse cancer cachexia models.

**Methods:**

The effects of anti‐GDF‐15 monoclonal antibody alone and in combination with an anti‐myostatin antibody or anamorelin fumarate were examined in GDF‐15‐dependent (HT‐1080 and RENCA) and partially dependent (TOV21G) mouse tumour models. Comprehensive assessments included food intake, body weight, body composition (including fat, lean and muscle mass), muscle function and treadmill running. Circulating myostatin was measured in patient samples from an advanced NSCLC clinical study.

**Results:**

Anti‐myostatin antibody treatment had limited efficacy in improving cachexia in mouse tumour models with high circulating GDF‐15 (HT‐1080 and RENCA), but improved cachexia (when combined with anti‐GDF‐15 antibody) in a tumour model with low circulating GDF‐15 levels (TOV21G). In the TOV21G model, combining anti‐myostatin and anti‐GDF‐15 antibodies led to even greater increases in body weight and hindlimb muscle mass compared with anti‐GDF‐15 antibody alone (*p* < 0.001 for muscle mass); however, the increase in muscle strength and treadmill running did not reach statistical significance over monotherapy. When anamorelin was combined with anti‐GDF‐15 antibody, body weight was elevated compared with the HT‐1080 tumour‐bearing vehicle group (*p* < 0.0001) but did not reach statistical significance over anti‐GDF‐15 antibody alone. Similar observations of the combination treatment were found for food intake, fat mass and gastrocnemius (*p* < 0.05). Circulating myostatin was negatively correlated with weight loss in patients with cancer (*p* < 0.01).

**Conclusion:**

These data provide proof‐of‐principle that mechanistically distinct approaches targeting muscle anabolism and appetite may act additively with GDF‐15 neutralization, particularly in cancer cachexia settings with lower GDF‐15 dependence.

**Trial Registration:** ClinicalTrials.gov identifier: NCT01360554.

## 1 Introduction

Cancer cachexia is a complex metabolic syndrome characterized by inflammation and involuntary weight loss driven by progressive depletion of skeletal muscle and adipose tissue. This tissue loss is thought to arise from a persistent energy imbalance, resulting from reduced food intake combined with a shift from anabolic to catabolic metabolism [1]. Currently, no pharmacologic treatment is approved by the regulatory authorities in the United States or EU for cancer cachexia [2]. A recent Phase 2 trial in patients with cancer cachexia (non–small‐cell lung cancer [NSCLC], colorectal and pancreatic cancer) and elevated circulating GDF‐15, the anti‐GDF‐15 antibody ponsegromab resulted in increased body weight, appetite, muscle mass and physical activity compared with placebo [3]. This study provides compelling evidence that targeting the GDF‐15 pathway may offer a viable therapeutic strategy, while raising new mechanistic questions about how GDF‐15 neutralization could be optimally integrated with other interventions to reverse the multifactorial cachexia syndrome.

In a chemotherapy‐induced cachexia rodent model, the combination of an anti‐GDF‐15 antibody and a melanocortin‐4 receptor antagonist (appetite stimulant) enhanced anti‐cachexia efficacy (food intake, body weight and fat mass) compared with anti‐GDF‐15 antibody monotherapy [4]. Similarly, combining the appetite stimulant anamorelin (ghrelin receptor agonist) with an ActRII pathway inhibitor resulted in greater efficacy for increased body weight/composition when given in combination in mouse models of lung cancer cachexia [5, 6]. Plasma GDF‐15 elevation was modest in the KL (Kras^LSL‐G12D/+^;Lkb1^flox/flox^) model, and the anti‐GDF‐15 antibody did not have an effect [5]. These data highlight the increased value of combining mechanisms that increase food intake and muscle anabolism.

The TGF‐β family members myostatin (GDF‐8), activin A and GDF‐11 are well‐established negative regulators of skeletal muscle mass across species [7]. Specifically, the ActRII–myostatin–activin A signaling pathway is one of the best validated molecular pathways driving skeletal muscle wasting in cancer cachexia, acting through SMAD2/3‐dependent repression of protein synthesis and activation of proteolytic systems [7]. The ghrelin axis is also a biologically compelling target: Endogenous ghrelin stimulates appetite, enhances gastric motility, promotes lipogenesis and attenuates catabolic and inflammatory signaling [8]. The clinical development experience targeting these pathways is extensive but disappointing. For example, ActRII receptor antagonists, the most potent approach for inhibiting this pathway, increased lean body mass but with no consistent improvement in physical function, strength or patient‐centred outcomes [9]. Similarly, anamorelin, a ghrelin receptor agonist, demonstrated consistent effects on appetite stimulation and increases in lean and fat mass across clinical studies, although again without improvements in physical function [10]. Together, these findings support a biological rationale for continued investigation of both the ActRII and ghrelin pathways as part of a comprehensive strategy to mitigate the metabolic and functional decline associated with cancer cachexia.

Consistent with the efficacy of ponsegromab in the basket (NSCLC, colorectal and pancreatic cancer) clinical trial [3], circulating GDF‐15 is associated with weight loss across numerous tumour types, including NSCLC and colorectal cancer [11, 12, 13, 14, 15, 16]. Circulating myostatin is also correlated with body weight change in patients with colorectal or lung cancer [17, 18], while activin A is associated with body weight loss primarily in lung and gastrointestinal cancer [11, 12, 17]. Since neutralizing activin A alone has not been demonstrated to yield a clear clinical benefit in cachexia [9], it is important to examine the relationship between myostatin, activin A, GDF‐11 and cancer cachexia in a GDF‐15‐validated sample set in which circulating GDF‐15 levels and their association with cachexia have been independently confirmed.

Because GDF‐15 neutralization alone increases both food intake and muscle mass, the objective of this series of studies was to determine whether combining an anti‐GDF‐15 antibody with either a muscle‐anabolic agent (an anti‐myostatin antibody) or an appetite stimulant (the ghrelin receptor agonist anamorelin) would produce superior outcomes in mouse models of cancer cachexia. An anti‐myostatin antibody was selected as the mechanistic tool to induce muscle anabolism because mice predominantly depend on myostatin, rather than activin A, as the primary negative regulator of muscle mass via Activin Receptor Type IIB (ActRIIB) signaling [19]. Furthermore, a selective anti‐activin A antibody suitable for in vivo use is not commercially available.

## 2 Methods

### 2.1 Experimental Models and Subject Details

Specific mouse strains and housing conditions as well as detailed methodology related to the generation of cell cultures and tumour models (GDF‐15‐dependent [HT‐1080 and RENCA] and partially dependent [TOV21G]) are provided in the Supporting Information.

All procedures were approved by the Pfizer Cambridge Animal Care and Use Committee (Animal Use Protocols KSQ‐01127), in accordance with the ethical standard laid down in the 1964 Declaration of Helsinki and its later amendments.

### 2.2 Treatments

Treatment with immunoglobulin G (IgG) control, anti‐GDF‐15 monoclonal antibody, anti‐myostatin antibody or ghrelin receptor agonist (anamorelin fumarate) was initiated as defined in each experiment (either in prevention or intervention mode when tumour‐bearing mice had ~10% average body weight loss from the animal's baseline). IgG control, anti‐GDF‐15 antibodies (10 mg/kg) and anti‐myostatin antibody (30 mg/kg) were generated by Pfizer and administered via subcutaneous injection once every 3 days (except in the RENCA and TOV21G tumour models, where it was administered once per week). Dosing volume was scaled to the weight of each individual mouse. The site of dosing was contralateral to the tumour. Dose selection of anti‐GDF‐15 antibody and anti‐myostatin antibody was based on previous publications [16, 20, 21].

Anamorelin fumarate (CAS 339539‐92‐3; BOC Sciences, Shirley, NY, USA) was dissolved in sterile phosphate‐buffered saline (PBS) (30 mg/kg), and the dosing volume was scaled according to the weight of each individual animal and administered once daily via oral gavage, whereas vehicle groups received PBS. The dose of anamorelin fumarate was selected based on previous publications [5, 22, 23, 24].

Fc‐GDF‐15 (GDF‐15 optimized in an Fc‐fusion protein format for improved half‐life extension) was generated by Pfizer and prepared in PBS and administered via subcutaneous injection as a single 0.1‐mg/kg dose. Recombinant human GDF‐15 (Cat# 120‐28; Peprotech, Cranbury, NJ, USA) was prepared in saline and administered via subcutaneous injection as a single dose of 0.1, 0.01 or 0.001 mg/kg.

### 2.3 Body Weight and Muscle Weight

Body weight was measured using a digital scale (measured between ~8 and 10 a.m.). Body weight for tumour‐bearing animals was calculated as body weight minus the weight of the tumour. At the end of the study, animals were euthanized, and gastrocnemius with or without soleus, tibialis anterior (TA) with or without extensor digitorum longus (EDL) and quadriceps muscles were isolated and weighed. Hindlimb muscle mass was calculated as the sum of these muscles as indicated in Section 3.

### 2.4 Body Composition Assessment

A whole‐body composition (fat mass, lean mass, free water and total water) analysis was performed using an EchoMRI (Houston, TX, USA) 4‐in‐1‐1100 on each animal 1 day prior to their euthanasia. Each mouse was individually placed in a specialized plastic tube for mild conscious restraint and inserted into a magnetic resonance imager (MRI) to measure body composition. Each mouse took ~2–3 min to complete the procedure. Mice were then returned to their home cages. Lean mass was determined for all animals by subtracting the tumour mass (tumour‐free lean mass) at the time of scanning, as tumour cell line implantation induces the growth of a solid subcutaneous tumour, and tumour tissue is likely considered as lean tissue when measured by MRI. No adjustment was performed for fat mass determination.

### 2.5 In Vivo Muscle Function Measurements

Mice were anesthetized with isoflurane and placed supine on a platform heated via a circulating water bath at 37°C. The right leg was shaved up to the patella and the right knee was stabilized via a knee clamp. Once stabilized, the right foot was affixed to a Dual Mode Foot Plate (300‐C FP; Aurora Scientific Inc., Aurora, Canada), and two electrodes were placed subcutaneously near the mid‐belly of the gastrocnemius muscle to achieve plantar flexion. Electrical stimulation was delivered (1 Hz, 0.2‐s duration and 1 s between stimulations) via stimulator (701C, Aurora Scientific) while increasing amperes to generate a maximum twitch measurement. After a maximum twitch was established, a force frequency of isometric contractions was initiated (0.2‐s duration and 120 s between stimulations). All data were collected and analysed using the manufacturer‐supplied software (Dynamic Muscle Control and Dynamic Muscle Analysis, Aurora Scientific).

### 2.6 Treadmill

Treadmill performance was characterized at the end of the study by measuring running time, distance and work using a motorized treadmill (Columbus Instruments, Columbus, OH, USA). Mice were trained daily on the treadmill for three consecutive weeks following treatment start for a duration of 20 min, a speed of 10 m/min and a grade of 5%. At study end, a treadmill endurance test was performed where animals ran on the treadmill at an initial speed and grade of 5 m/min and 5% for 2 min, then 7 m/min for 2 min, 10 m/min for 2 min and, finally, at every subsequent 2 min, the speed was increased by 2 m/min until the mice were exhausted. Exhaustion was defined as the mouse falling off the treadmill onto an electrical shocker three times within 15 s. Running time, speed and distance were recorded, and work (the product of body weight [kg], gravity [9.81 m/s^2^], vertical speed [m/s 3 angle] and time [s]) was calculated. The treadmill protocol used in this study aligns with current standard practice in the field [5].

### 2.7 Food Intake and Pair‐Feeding Study

For food intake measurement, animals were housed individually. For tumour model studies, food pellets were weighed and placed each day in a ceramic bowl on the bottom of the cage. On the next day, the food pellets from the previous day were reweighed and discarded. Any visible food pellets in the cage that were not consumed were picked up and weighed together with the pellets in the ceramic bowl to accurately measure daily food intake and account for food spillage. This method was used because the majority of crumbs are collected in the ceramic bowl, which greatly increases measurement accuracy. Daily food intake was calculated as the weight difference in food between two consecutive days, and the cumulative food intake was calculated as the sum of food intake over a defined period.

For the pair‐feeding study, starting on Day 0, food was restricted in a group of HT‐1080 tumour‐bearing mice receiving anti‐GDF‐15 antibody treatment to match the food intake and body weight of the isotype tumour‐bearing control group. Pair‐fed mice were given the amount of food consumed by the isotype‐treated mice from the previous day. Food was given within 6 h of the onset of the dark cycle each day in the ceramic bowls placed on the cage bottom.

### 2.8 GDF‐15, Myostatin, Activin A and GDF‐11 Measurement

Human GDF‐15 in plasma was measured using a human GDF‐15 Quantikine ELISA kit (catalogue: DGD150; R&D Systems, Minneapolis, MN, USA) following the manufacturer's instructions. Mouse GDF‐15 in plasma was measured using a mouse/rat GDF‐15 Quantikine ELISA kit (catalogue: MGD150) following the manufacturer's instructions. Human activin A in plasma was measured using a human/mouse/rat activin A Quantikine ELISA kit (catalogue: DAC00B). Human/mouse myostatin and human GDF‐11 were measured using an internal liquid chromatography–mass spectrometry assay.

### 2.9 ARCHER1009 Cohort

ARCHER1009 was a randomized Phase 3 study for patients with advanced NSCLC, who were randomized to the epidermal growth factor receptor inhibitors dacomitinib versus erlotinib [25]. This study received ethical approval from the Pfizer Institutional Review Board and was conducted in accordance with the Declaration of Helsinki (NCT01360554). For the cachexia study, 164 patients with available plasma and body weight data at two timepoints within 6 months were included. Classification of weight loss was similar to published schemes [1] and defined as either weight stable/gain, > 0%–5% weight loss or > 5% weight loss. Any prior treatment (chemotherapy, radiation or surgery) must have been completed at least 2 weeks prior to randomization at the start of the study.

Body weight data were collected from medical records in routine healthcare settings; scale calibrations and type of clothing were conducted according to local standards. Body weight changes were assessed between time of enrollment and end of study. Blood samples were obtained at the end of study for the measurement of myostatin, activin A and GDF‐11.

### 2.10 Quantification and Statistical Analysis

Detailed statistical methods are provided in the Supporting Information.

## 3 Results

### 3.1 Anti‐Myostatin Antibody Treatment Has Limited Efficacy to Improve Cachexia in Mouse Tumour Models With High Circulating GDF‐15 Concentrations

HT‐1080 and RENCA mouse tumour models were selected because they are each associated with high circulating GDF‐15 concentrations and GDF‐15‐dependent cachexia but represent different tumour types and mouse strain backgrounds.

#### 3.1.1 HT‐1080

**FIGURE 1:**
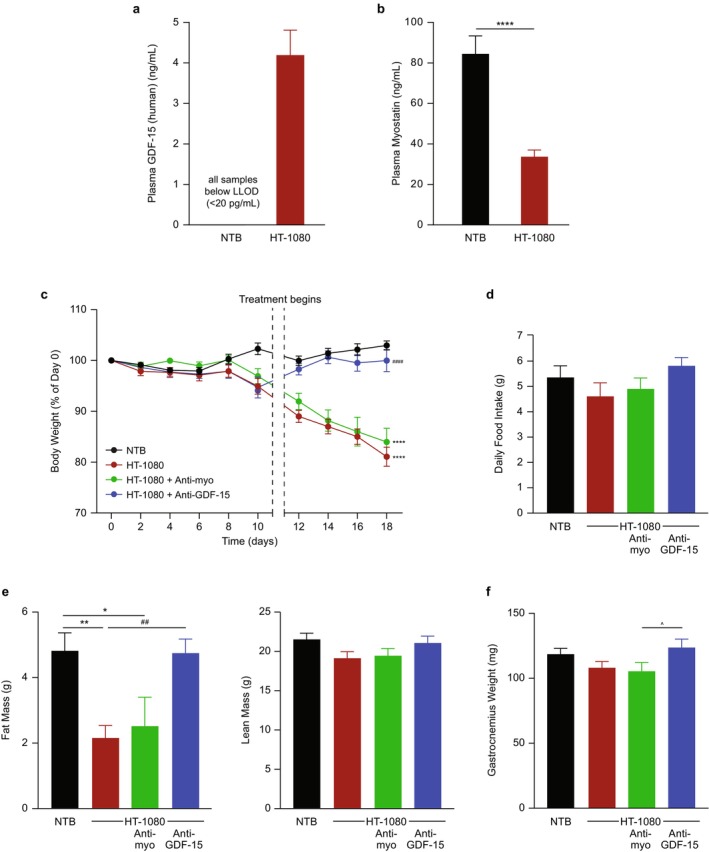
Anti‐myostatin antibody treatment has limited efficacy to improve cachexia, when given alone, in a tumour model (HT‐1080) with high circulating GDF‐15 elevation. (a, b) Circulating plasma levels of (a) GDF‐15 and (b) myostatin in NTB and HT‐1080 tumour‐bearing mice at the end of study (*n* = 8–10 per group). (c) Tumour‐free body weight percentage change in NTB and HT‐1080 tumour‐bearing mice treated with either control IgG, anti‐GDF‐15 antibody or anti‐myostatin antibody during the study (*n* = 9–10 per group). (d) Daily food intake at the end of study (*n* = 8–10 per group). (e, f) Body composition, including (e) fat and lean mass and (f) gastrocnemius weight at the end of study (*n* = 9–10 per group). Data are mean ± SEM. *p < 0.05, **p < 0.01, ****p < 0.0001 vs. NTB; ^##^ *p* < 0.01, ^####^ *p* < 0.0001 vs. HT‐1080; ^*p* < 0.05 vs. HT‐1080 + anti‐myostatin. GDF‐15, growth differentiation factor‐15; IgG, immunoglobulin G; LLOD, lower limit of detection; NTB, non–tumour‐bearing; SEM, standard error of the mean.

The HT‐1080 tumour model generated a cachectic phenotype characterized by increases in plasma GDF‐15 (Figure 1a), decreases in plasma myostatin levels (*p* < 0.0001; Figure 1b), progressive weight loss (*p* < 0.0001; Figure 1c) and reductions in fat mass (*p* < 0.01; Figure 1e), consistent with previous studies [22]. Modest effects were observed on daily food intake, lean mass and gastrocnemius weight, but these did not reach statistical significance relative to respective vehicle groups, which is likely due to the limited duration of the model determined by aggressive tumour growth (Figure 1d–f). Neutralization of GDF‐15 with the anti‐GDF‐15 antibody significantly reversed body weight loss (*p* < 0.0001), restored fat mass (*p* < 0.01) and increased gastrocnemius weight (*p* < 0.05) in tumour‐bearing mice (Figure 1c,e,f), whereas treatment with the anti‐myostatin antibody had negligible effects (Figure 1c,e,f). There were no statistically significant differences between any treatment groups for tumour weight (Table S1).

**FIGURE 2:**
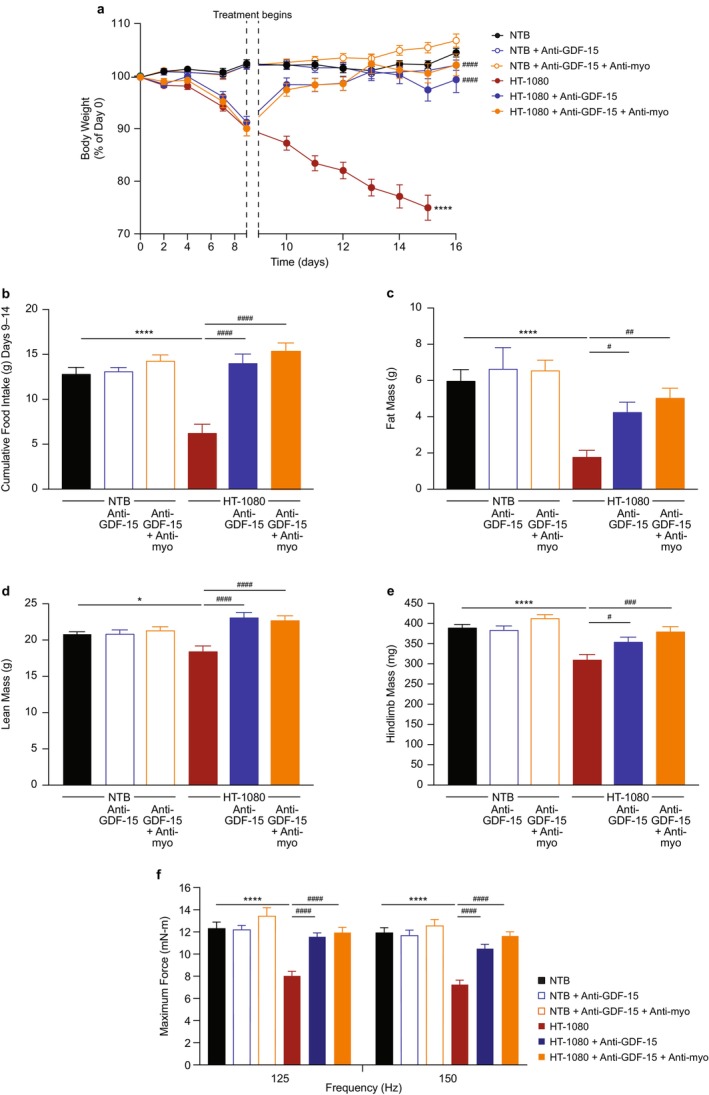
Anti‐myostatin antibody treatment has limited efficacy to improve cachexia, when given alone or in combination with anti‐GDF‐15 antibody, in a tumour model (HT‐1080) with high circulating GDF‐15 elevation. (a) Tumour‐free body weight percentage change in NTB and HT‐1080 tumour‐bearing mice treated with either control IgG, anti‐GDF‐15 antibody or the combination of anti‐GDF‐15 antibody and anti‐myostatin antibody during the study. (b) Cumulative food intake during the treatment period (Days 9–14). (c–e): Body composition, including (c) fat mass, (d) lean mass and (e) hindlimb muscle mass at the end of study. (f) Maximum force at 125 and 150 Hz in the muscle function test. *N* = 12–16 per group for a–f. Data are mean ± SEM. *p < 0.05, ****p < 0.0001 vs. NTB; ^#^ *p* < 0.05; ^##^ *p* < 0.01, ^###^ *p* < 0.001; ^####^ *p* < 0.0001 vs. HT‐1080. GDF‐15, growth differentiation factor‐15; IgG, immunoglobulin G; NTB, non–tumour‐bearing; SEM, standard error of the mean.

The effects of anti‐GDF‐15 antibody in combination with anti‐myostatin antibody were evaluated in a separate study in non–tumour‐bearing and HT‐1080 tumour‐bearing mice (Figure 2 and Table S1). In this experiment, there was greater weight loss and food intake reduction compared to Figure 1, likely due to the larger tumour size/disease progression. Treatment with anti‐GDF‐15 antibody significantly increased measures of body weight (*p* < 0.0001), cumulative food intake (Days 9–14; *p* < 0.0001), body composition (fat, lean and hindlimb muscle mass; *p* < 0.05), tumour weight (*p* < 0.01) and muscle function (*p* < 0.0001) (Figure 2a–f and Table S2). The hindlimb muscle mass improvement included increases for the gastrocnemius/soleus complex (*p* < 0.01) and quadriceps (*p* < 0.05) and a trend for the TA/EDL (Table S2). The addition of anti‐myostatin antibody resulted in some increases of these measures beyond the effects observed with anti‐GDF‐15 antibody alone, but these differences were not statistically significant (Figure 2a–f and Tables S1 and S2).

To determine whether the increased food intake induced by treatment with the anti‐GDF‐15 antibody is the primary driver of the beneficial effects on body weight and body composition, pair‐feeding was applied to HT‐1080 tumour‐bearing mice also receiving the anti‐GDF‐15 antibody to match the food intake and body weight of the isotype control group. The effect of anti‐GDF‐15 antibody on body weight, lean mass, fat mass and tumour weight (Figure S1) was completely attenuated in mice on a calorie‐restricted diet (*p* < 0.0001), except for a small portion of the fat mass gain. These data suggest the increased HT‐1080 tumour growth in the anti‐GDF‐15 antibody group is an indirect effect and secondary to the increase in food intake. These results also highlight the importance of food intake on the beneficial effects of GDF‐15 neutralization.

#### 3.1.2 RENCA

**FIGURE 3:**
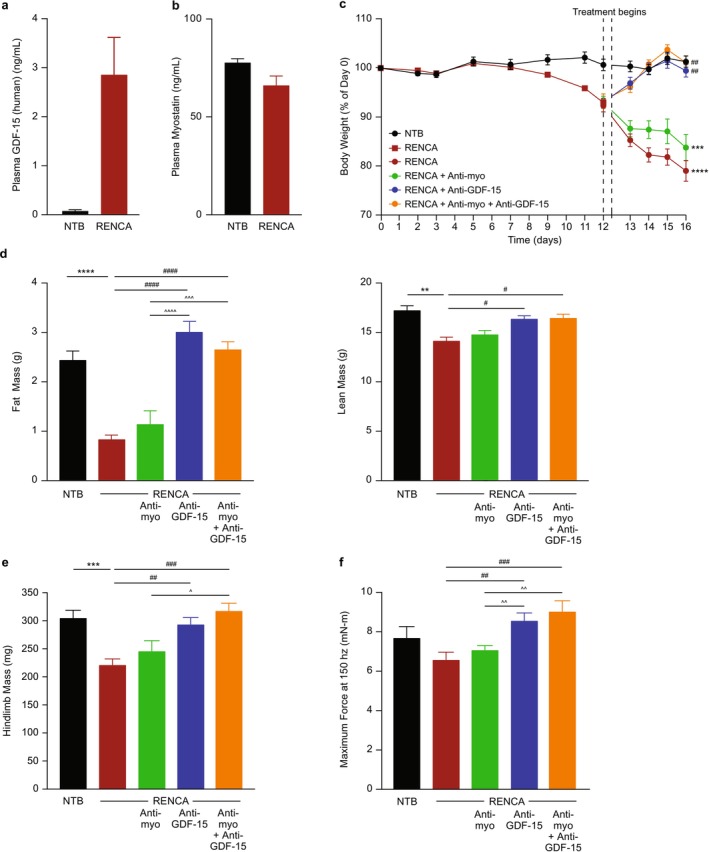
Anti‐myostatin antibody treatment has limited efficacy to improve cachexia when given alone or in combination with anti‐GDF‐15 antibody in a mouse tumour model (RENCA) with high circulating GDF‐15 elevation. (a, b) Circulating plasma levels of (a) GDF‐15 and (b) myostatin in NTB and RENCA tumour‐bearing mice at the end of study (*n* = 5–10 for NTB; *n* = 11 for RENCA). (c) Tumour‐free body weight percentage change in NTB and RENCA tumour‐bearing mice treated with control IgG, anti‐myostatin antibody or anti‐GDF‐15 antibody alone or in combination with anti‐myostatin antibody during the study. (d, e) Body composition, including (d) lean and fat mass and (e) hindlimb muscle mass at the end of study. (f) Maximum force at 150 Hz in the muscle function test. *n* = 8–16 per group for c–f. Data are mean ± SEM. **p < 0.01, ***p < 0.001, ****p < 0.0001 vs. NTB; ^#^ *p* < 0.05; ^##^ *p* < 0.01, ^###^ *p* < 0.001; ^####^ *p* < 0.0001 vs. RENCA; ^*p* < 0.05, ^^*p* < 0.01, ^^^p < 0.001, ^^^^*p* < 0.0001, for all other comparisons between RENCA groups treated with anti‐myostatin and/or anti‐GDF‐15. GDF‐15, growth differentiation factor‐15; IgG, immunoglobulin G; NTB, non–tumour‐bearing; SEM, standard error of the mean.

The combination treatment was evaluated in a second mouse model of cancer cachexia, RENCA, that demonstrated increases in plasma GDF‐15 comparable to the HT‐1080 mouse model (Figure 3a and Tables S1 and S2). The model was associated with a mild trend for reduced plasma myostatin (Figure 3b), increased plasma activin A (NTB: 0.10 ± 0.01 ng/mL; tumour: 0.30 ± 0.05 ng/mL; *p* < 0.05), significant progressive body weight loss (*p* < 0.0001; Figure 3c) that included lean, fat and hindlimb muscle mass (*p* < 0.01; Figure 3d,e). The hindlimb muscle mass reduction included the gastrocnemius/soleus complex (*p* < 0.01) and quadriceps (*p* < 0.05) and a trend for the TA/EDL (Table S2). Treatment with anti‐GDF‐15 antibody significantly reversed reductions in body weight and measures of body composition (*p* < 0.05; Figure 3c–e and Table S2) and improved muscle function (*p* < 0.01; Figure 3f). Anti‐myostatin antibody by itself and in combination with anti‐GDF‐15 antibody resulted in modest improvements across these measures that did not reach statistical significance (vs. RENCA + IgG and RENCA + anti‐GDF‐15, respectively) (Figure 3c–f and Table S2). There were no statistically significant differences between any treatment groups for tumour weight (Table S1).

### 3.2 Anti‐Myostatin Antibody Treatment Improves Cachexia in a Tumour Model (TOV21G) With Low Circulating GDF‐15 Concentrations

**FIGURE 4:**
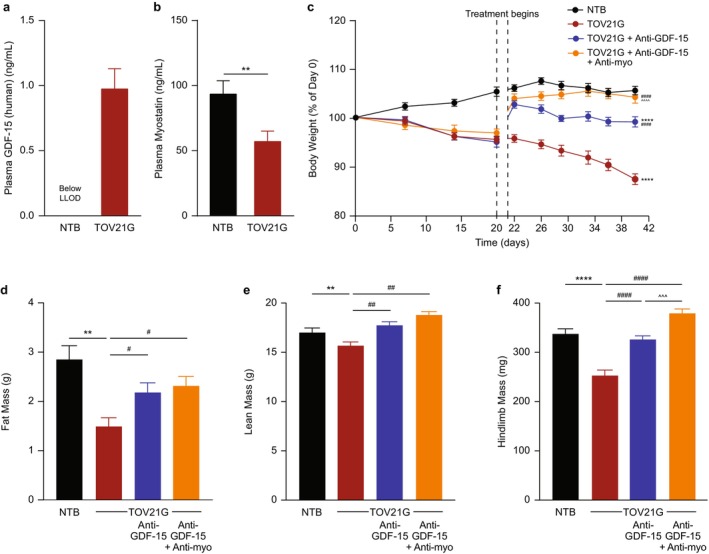
Anti‐myostatin antibody treatment improves cachexia when given alone or in combination with anti‐GDF‐15 antibody in a tumour model (TOV21G) with low circulating GDF‐15 elevation. (a, b) Circulating plasma levels of (a) GDF‐15 and (b) myostatin in NTB and TOV21G tumour‐bearing mice at the end of study (*n =* 8 per group). (c) Tumour‐free body weight percentage change in NTB and TOV21G tumour‐bearing mice treated with control IgG or anti‐GDF‐15 antibody alone or in combination with anti‐myostatin antibody during the study. (d–f) Body composition, including (d) fat mass, (e) lean mass and (f) hindlimb muscle mass at the end of study. *n* = 15–17 per group for c–f. Data are mean ± SEM. **p < 0.01, ****p < 0.0001 vs. NTB; ^#^ *p* < 0.05, ^##^ *p* < 0.01, ^####^ *p* < 0.0001 vs. TOV21G; ^^^*p* < 0.001, ^^^^*p* < 0.0001, for comparison between TOV21G + anti‐GDF‐15 and TOV21G + anti‐GDF‐15 + anti‐myostatin. GDF‐15, growth differentiation factor‐15; IgG, immunoglobulin G; LLOD, lower limit of detection; NTB, non–tumour‐bearing; SEM, standard error of the mean.

**FIGURE 5:**
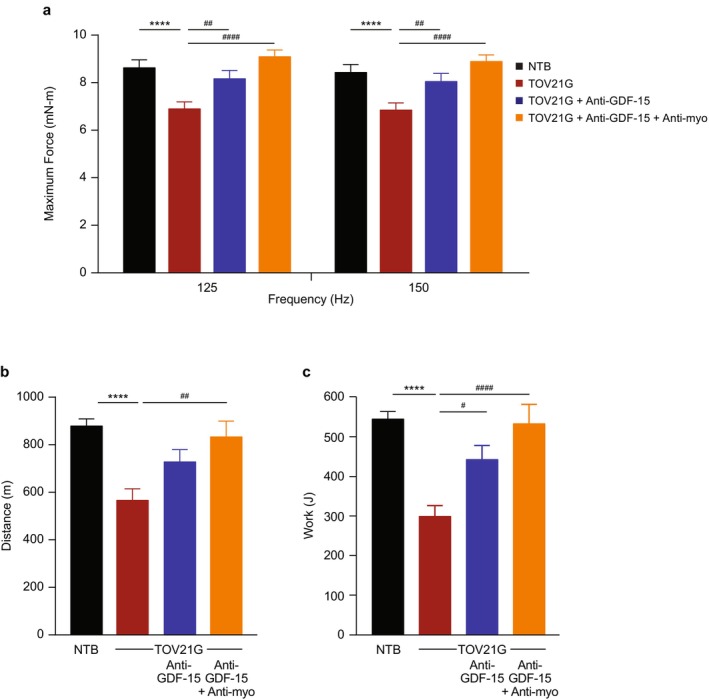
Anti‐myostatin antibody treatment improves muscle function when given alone or in combination with anti‐GDF‐15 antibody in a tumour model (TOV21G) with low circulating GDF‐15 elevation. (a) Maximum force at 125 and 150 Hz in the muscle function test. (b, c) A treadmill endurance test was conducted at the end of the study with (b) running distance measured and (c) work calculated accounting for the effect of body weight on treadmill performance. *N* = 15–17 per group for a–c. Data are mean ± SEM. ****p < 0.0001 vs. NTB; ^#^ *p* < 0.05, ^##^ *p* < 0.01, ^####^ *p* < 0.0001 vs. TOV21G. GDF‐15, growth differentiation factor‐15; NTB, non–tumour‐bearing; SEM, standard error of the mean.

The TOV21G mouse tumour model was also examined, as it has lower circulating GDF‐15 concentrations (Figure 4a) relative to the HT‐1080 and RENCA models and is only partially GDF‐15‐dependent [20], which aids in optimizing the sensitivity window to detect efficacy. As in the previous tumour models, tumour‐bearing mice showed significantly decreased myostatin levels (*p* < 0.01; Figure 4b); progressive weight loss (*p* < 0.0001; Figure 4c); and reductions in fat, lean and hindlimb muscle mass (*p* < 0.01; Figure 4d–f and Table S2). The hindlimb muscle mass reduction included the gastrocnemius/soleus complex (*p* < 0.0001), TA (*p* < 0.01) and quadriceps (*p* < 0.0001) (Table S2). There were no statistically significant differences between any treatment groups for tumour weight (Table S1). Muscle function, including muscle strength (maximum force) and treadmill running (distance and work), was also assessed with significant impairment observed in tumour‐bearing mice (*p* < 0.0001; Figure 5a–c).

Treatment with anti‐GDF‐15 antibody resulted in significant improvements in body weight (*p* < 0.0001; Figure 4c); increases in fat, lean and hindlimb muscle mass (including gastrocnemius/soleus complex, TA and quadriceps) (*p* < 0.05; Figure 4d–f and Table S2); and improved muscle strength and treadmill running (*p* < 0.05; Figure 5a,c). Combining treatment with anti‐myostatin antibody provided even greater increases in body weight and hindlimb muscle mass (including TA and quadriceps, and trend for gastrocnemius/soleus complex) than anti‐GDF‐15 antibody alone (*p* < 0.001; Figure 4c,f and Table S2) and raised muscle strength and treadmill running to healthy non–tumour‐bearing levels, although the effect compared with anti‐GDF‐15 antibody monotherapy did not reach statistical significance (Figure 5a–c).

### 3.3 The Effect of Anti‐Myostatin Antibody Is Decreased by the Presence of GDF‐15 in Healthy Mice

**FIGURE 6:**
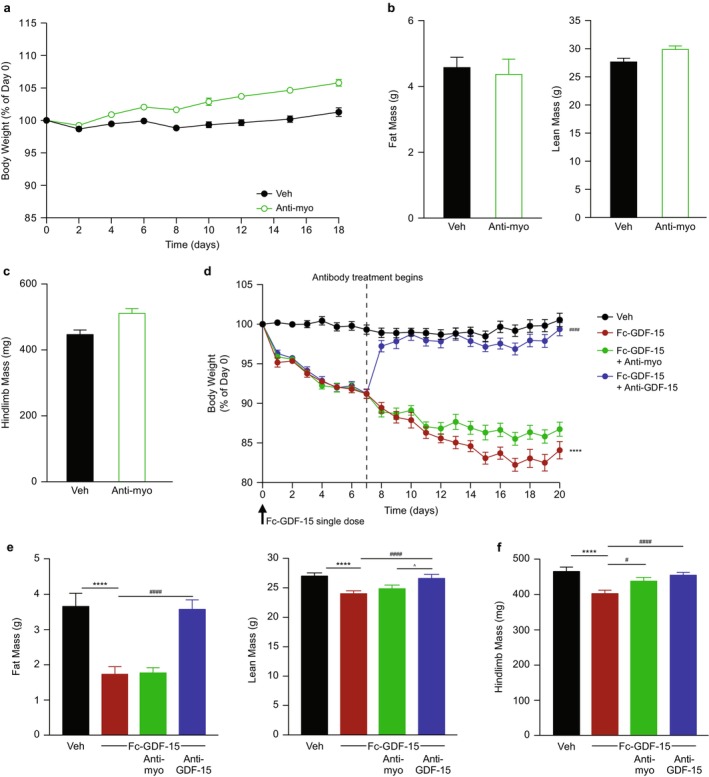
The effect of anti‐myostatin antibody is decreased by the presence of GDF‐15 in healthy mice. (a) Body weight percentage change in healthy mice treated with control IgG (Veh) or anti‐myostatin antibody during the study. (b, c) Body composition, including (b) fat and lean mass and (c) hindlimb muscle mass at the end of study in healthy mice. (d) Body weight percentage change in healthy mice treated with Fc‐GDF‐15 alone (with control IgG) or in the presence of anti‐GDF‐15 antibody or anti‐myostatin antibody. Veh control received PBS and IgG. (e, f) Body composition, including (e) fat and lean mass and (f) hindlimb muscle mass at the end of study in healthy mice treated with vehicle or Fc‐GDF‐15 alone or in the presence of anti‐GDF‐15 or anti‐myostatin antibody. *N* = 15 per group for a–f. Data are mean ± SEM. ****p < 0.0001 vs. Veh; ^#^ *p* < 0.05, ^####^ *p* < 0.0001 vs. Fc‐GDF‐15; ^*p* < 0.05, Fc‐GDF‐15 + anti‐myostatin vs. Fc‐GDF‐15 + anti‐GDF‐15. GDF‐15, growth differentiation factor‐15; IgG, immunoglobulin G; PBS, phosphate‐buffered saline; SEM, standard error of the mean; Veh, vehicle.

The interaction between myostatin and GDF‐15 on body weight and body composition was evaluated in healthy mice. Treatment with anti‐myostatin antibody was confirmed to increase progressive body weight and lean and hindlimb muscle mass (including gastrocnemius/soleus complex, TA/EDL and quadriceps) (Figure 6a–c and Table S2). Fc‐GDF‐15 was associated with progressive body weight loss and significant decreases in fat, lean and hindlimb muscle mass (all muscle types), each of which was reversed by anti‐GDF‐15 antibody (*p* < 0.0001; Figure 6d–f and Table S2). In contrast, when treatment with anti‐myostatin antibody was combined with Fc‐GDF‐15, only a modest increase in body weight was observed (Figure 6d), with minimal effects on body composition, although significant improvement was demonstrated on hindlimb muscle mass (*p* < 0.05; Figure 6e,f and Table S2). The hindlimb muscle mass increase included quadriceps (*p* < 0.05) and trends for gastrocnemius/soleus complex and TA/EDL (Table S2). When comparing the two studies, the anti‐myostatin antibody effect on body weight (Figure 6a: 4.5% difference between Veh vs. anti‐myostatin; Figure 6d: 2.6% difference Fc‐GDF‐15 vs. Fc‐GDF‐15 + anti‐myostatin) and hindlimb mass (Figure 6c: 64.6‐mg difference between Veh vs. anti‐myostatin; Figure 6f: 35.4‐mg difference Fc‐GDF‐15 vs. Fc‐GDF‐15 + anti‐myostatin) was diminished when GDF‐15 was present.

### 3.4 Ghrelin Receptor Agonism Improves Cachexia When Given Alone or in Combination With Anti‐GDF‐15 Antibody in the HT‐1080 Tumour Model

**FIGURE 7:**
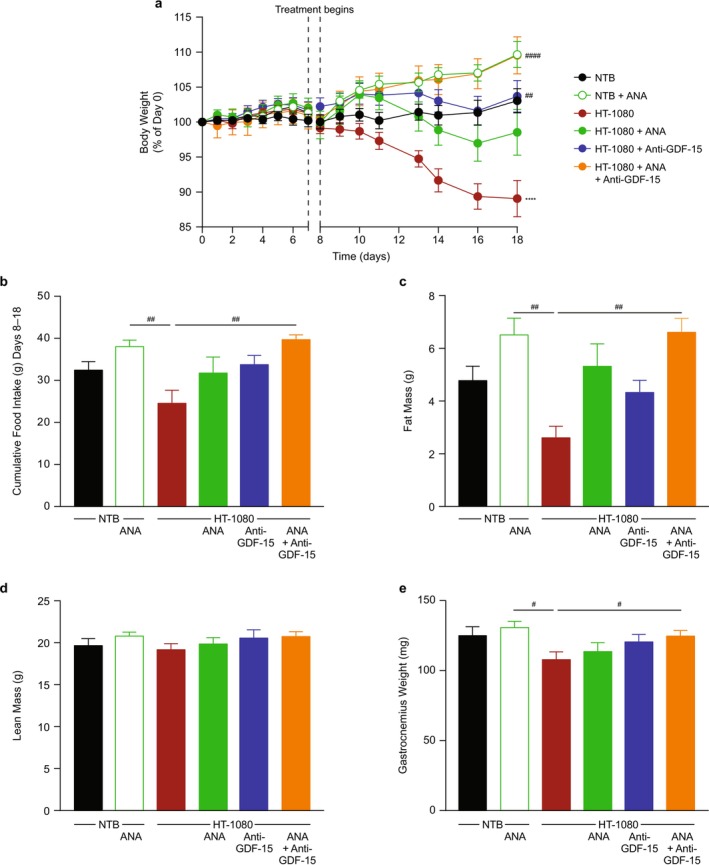
Ghrelin receptor agonism (anamorelin) improves cachexia when given alone or in combination with anti‐GDF‐15 antibody in a tumour model (HT‐1080) with high circulating GDF‐15 elevation. (a) Tumour‐free body weight percentage change in NTB and HT‐1080 tumour‐bearing mice treated with vehicle (PBS) or anamorelin in the presence or absence (IgG control) of anti‐GDF‐15 antibody (*n* = 9–12 per group). (b) Cumulative food intake during the treatment period (Days 8–18) (*n* = 7–10 per group). (c–e) Body composition, including (c) fat and (d) lean mass and (e) gastrocnemius weight at the end of study (*n* = 9–12 per group). Data are mean ± SEM. ****p < 0.0001 vs. NTB; ^#^ *p* < 0.05, ^##^ *p* < 0.01, ^####^ *p* < 0.0001 vs. HT‐1080. GDF‐15, growth differentiation factor‐15; IgG, immunoglobulin G; NTB, non–tumour‐bearing; PBS, phosphate‐buffered saline; SEM, standard error of the mean.

The ghrelin receptor agonist anamorelin increased body weight over time in both non–tumour‐bearing and HT‐1080 tumour‐bearing mice compared with the corresponding vehicle groups (NTB, HT‐1080) (Figure 7a). When anamorelin was combined with anti‐GDF‐15 antibody, body weight was elevated compared with anti‐GDF‐15 alone in tumour‐bearing mice (Figure 7a). Similar benefits of the combination treatment were observed for food intake, fat mass and gastrocnemius weight, where significant increases (relative to the HT‐1080 group) were shown only when anamorelin was given in combination with anti‐GDF‐15 antibody in tumour‐bearing mice (*p* < 0.05; Figure 7b,c,e). There were no significant treatment effects on lean mass (Figure 7d) or terminal tumour weight (Table S1).

The interaction between ghrelin receptor agonism and GDF‐15 on food intake was evaluated in healthy mice. Treatment with anamorelin was confirmed to increase acute food intake at 6 and 24 h after treatment (*p* < 0.0001; Figure S2). The benefit of anamorelin on food intake was decreased with increasing doses of exogenously administered GDF‐15 (*p* < 0.05; Figure S2).

### 3.5 Circulating Myostatin Is Associated With Weight Loss in Patients With Cancer

**FIGURE 8:**
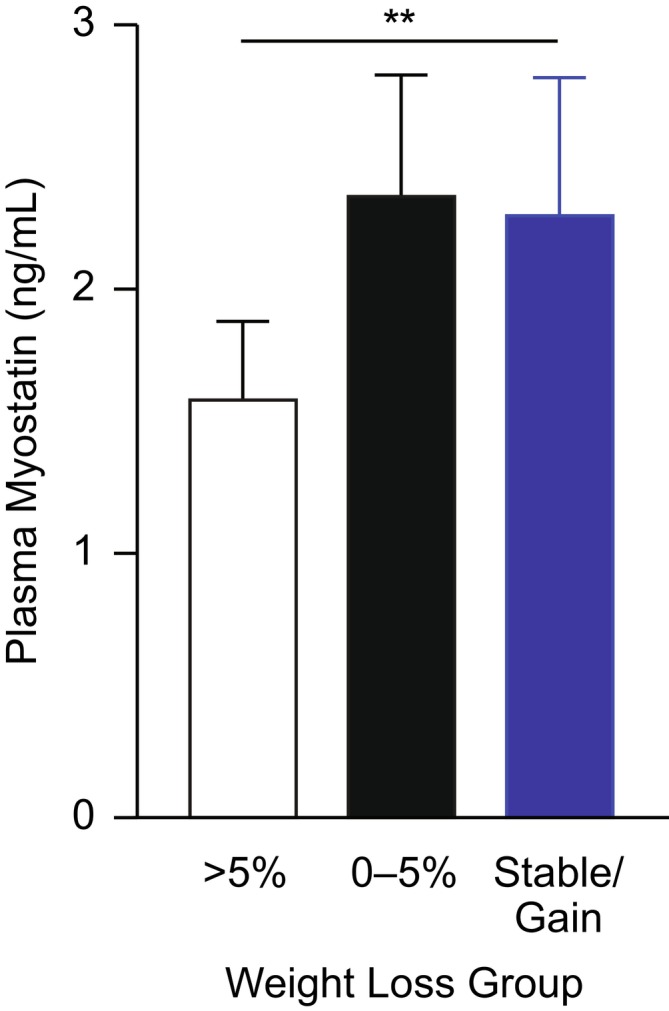
Circulating plasma levels of myostatin and their association with the degree of body weight loss in individuals with advanced NSCLC. Data were obtained from the ARCHER1009 study [25] and show myostatin levels according to weight loss group (*n* = 63 for > 5%; *n* = 58 for 0%–5%; *n* = 43 for Stable/Gain [patients who demonstrated stable weight or weight gain]). Data are geometric mean and 95% confidence intervals. **p < 0.01 vs. Stable/Gain. NSCLC, non–small‐cell lung cancer.

Consistent with the mechanistic questions addressed in the experimental models, circulating myostatin (as well as related ActRII ligands activin A and GDF‐11) was measured in a patient sample set where an association between GDF‐15 and weight loss in advanced NSCLC was previously reported [15, 25]. Unfortunately, a patient sample set also matching the tumour types of the mouse models could not be identified. Plasma myostatin demonstrated a significant relationship with body weight change, with concentrations significantly lower in individuals with > 5% weight loss compared with no weight loss (stable/weight gain) (*p* < 0.01; Figure 8). Activin A and GDF‐11 were not associated with body weight loss (Figure S3a,b). GDF‐11 has been implicated in muscle wasting/cachexia [26] and, interestingly, was suggested to decrease food intake by inducing GDF‐15 in rodent models [27]. In healthy mice, exogenous administration of GDF‐11 was associated with significant increases in plasma GDF‐15 and concomitant decreases in body weight (*p* < 0.0001; Figure S3c–f). However, the circulating GDF‐11 concentrations that were associated with weight loss in mice were considerably higher than those observed in patients with advanced NSCLC (mice: > 300 ng/mL; patients: < 1 ng/mL) (Figure S3).

## 4 Discussion

Recent Phase 2 data with the anti‐GDF‐15 antibody ponsegromab [3] support the hypothesis that GDF‐15 is a primary driver of cancer cachexia, improving appetite and body weight (including skeletal muscle mass), and establish the therapeutic potential of GDF‐15 neutralization for further evaluation in clinical trials. Clinical trials of other agents have not shown benefits sufficient for the US Food and Drug Administration regulatory approval; however, improvements in body composition were reported [28]. Taken together, this raises important mechanistic questions about how GDF‐15 neutralization interacts with other food intake and muscle anabolic pathways, and what mechanistic combinations with GDF‐15 neutralization could optimize efficacy for patients. Given the clinical relevance and availability of rodent‐appropriate tools, the objective of the series of studies reported herein was to evaluate the efficacy of anti‐GDF‐15 antibody treatment in combination with muscle anabolic (anti‐myostatin antibody) or appetite stimulant (ghrelin receptor agonist anamorelin) modulators using experimental mouse tumour models.

Since mice predominantly depend on myostatin as the primary negative regulator of ActRIIB‐dependent muscle mass [19], anti‐myostatin antibody was selected for the mechanistic combination experiments. The GDF‐15‐dependent HT‐1080 and RENCA mouse tumour models were selected because they have similar circulating GDF‐15 concentrations but represent different tumour types and mouse strain backgrounds. Anti‐myostatin antibody had modest efficacy as a monotherapy or when administered in combination with anti‐GDF‐15 antibody for body composition and weight and muscle function as assessed by maximum force generation. The greater hindlimb muscle mass in the NTB + anti‐GDF‐15 + anti‐myostatin antibody group compared with the HT‐1080 + anti‐GDF‐15 antibody group suggests the sensitivity window for detecting a benefit was sufficient and did not preclude detection of further muscle mass increases generated in the HT‐1080 + anti‐GDF‐15 + anti‐myostatin antibody combination group. The TOV21G mouse tumour model was also examined since it has a lower circulating GDF‐15 concentration and is partially GDF‐15‐dependent, which aids in optimizing the sensitivity window to detect efficacy. Anti‐myostatin antibody in combination with anti‐GDF‐15 antibody provided greater body weight and muscle mass increase than anti‐GDF‐15 antibody monotherapy. Muscle strength and treadmill running also showed greater benefit in the combination, raising muscle function to healthy non–tumour‐bearing levels. The differing responses between the HT‐1080/RENCA and TOV21G tumour models may be related to different GDF‐15 levels and other pro‐cachectic factors that impact skeletal muscle. A systematic evaluation of circulating pro‐cachexia biomarkers is not available; however, activin A has been reported to be similar in these models but with higher interleukin‐6 in the TOV21G model [12] (summarized in Table S3).

In support of the concept that GDF‐15 influences the myostatin pathway, in healthy mice, where only GDF‐15 was elevated, the benefit of anti‐myostatin antibody on body weight and composition was decreased, reinforcing the concept that patients with cancer cachexia with low GDF‐15 could have greater benefit from muscle anabolic therapy. Furthermore, the additive effect of these two mechanisms suggests the effect of GDF‐15 on muscle is independent of the ActRII pathway. Since published reports suggest blocking the ActRII receptor or multiple ligands (e.g., myostatin and activin A) results in greater muscle hypertrophy [29], future experiments are warranted to investigate this combination concept or with other muscle anabolic mechanisms (e.g., selective androgen receptor modulator) in cancer cachexia. Given that our clinical and experimental data suggest circulating myostatin is likely a biomarker of reduced muscle mass, and muscle biopsies for measurement of myostatin expression are not feasible in clinical trials, it may be challenging to identify which patients can expect the greatest benefit. Perhaps muscle anabolic therapies would be best utilized prior to patients developing refractory cachexia to prevent the loss of muscle.

The ghrelin pathway is a homeostatic mechanism that increases appetite and also growth hormone/insulin‐like growth factor‐1‐dependent muscle growth [30]. In the GDF‐15‐dependent HT‐1080 tumour model, anamorelin alone was effective to increase food intake and fat mass, but not muscle mass. Even with the improvements with the anti‐GDF‐15 antibody, anamorelin was able to further increase body weight, food intake and fat mass, illustrating additivity of these two mechanisms. These data also suggest the ability to detect improvements for cachexia endpoints in the HT‐1080 model was not saturated in the experiment with anti‐myostatin antibody. Similar to the observation with anti‐myostatin antibody, GDF‐15 also has a significant influence on the ghrelin pathway where, in healthy mice, when only GDF‐15 was elevated, the benefit of anamorelin on food intake was decreased. This supports the concept that cancer cachexia patients with low GDF‐15 could have greater benefit from ghrelin receptor agonism.

Neutralization of GDF‐15 function did not affect tumour weight in the RENCA and TOV21G tumour models (consistent with published work [12, 20]), but it increased tumour weight inconsistently in the HT‐1080 model in this study (unchanged in two experiments; increased in two experiments). No impact of GDF‐15 inhibition on HT‐1080 tumour growth was previously reported [12]. The pair‐feeding experiment supports that this is not a direct effect of GDF‐15 neutralization and is secondary to the increased food intake. The combination of results suggests that GDF‐15 monotherapy does not have a direct role in regulating tumour growth in these experimental models.

To assess circulating concentrations of the cachexia biomarkers of interest, GDF‐15 and key ligands of the ActRII signaling pathway, myostatin, activin A and GDF‐11 were measured in the mouse tumour models, and their association with the degree of body weight loss in patients with advanced NSCLC was also assessed. An association of GDF‐15 with weight loss was reported previously in the NSCLC sample set [15]. First, all biomarkers were within a consistent range across the mechanistic mouse tumour models investigated (HT‐1080, RENCA, TOV21G) (Table S3). Furthermore, mouse concentrations of GDF‐15, myostatin and GDF‐11 are consistent with the NSCLC patient samples (Figures 8 and S3) and also across the tumour types with published data available (summarized in Section 1). However, myostatin is approximately 30‐fold higher in mouse, because mice circulate more latent myostatin as a buffering pool, whereas humans keep circulating levels low and rely more on local, tissue‐restricted signaling [31].

For the clinical samples, opposite to GDF‐15, myostatin showed a positive relationship with body weight change, which is consistent with published reports in patients with colorectal or lung cancer [17, 18], and may suggest low circulating myostatin is a result of reduced muscle mass rather than a biomarker of ActRII receptor activation [17, 18, 32]. Interestingly, there is no evidence of a relationship between circulating activin A and body weight change in this sample set from patients with advanced NSCLC. Published reports for activin A are mixed showing an association with body weight loss in lung and gastrointestinal cancer [12, 17], and a study combining multiple tumour types [12] but not in some other studies in lung cancer [11] and also mixed tumour types [11]. More investigation is needed to address the global relevance of activin A signaling in cachexia across tumour types and syndrome stage. Like activin A, there was no association between circulating GDF‐11 and weight loss in patients with advanced NSCLC in the analysis presented herein. Consistent with the lack of association with weight loss, only circulating GDF‐11 concentrations that were significantly higher than the patient values caused weight loss in mice (patients: < 1 ng/mL; mice: > 300 ng/mL), which is unlike the pharmacologic relationship reported for GDF‐15 and activin A. These data do not support a role for GDF‐11 in cancer cachexia. Interestingly, consistent with the circulating biomarker data, a recent transcriptomic analysis of skeletal muscle from patients with pancreatic and colorectal cancer cachexia found no differential expression of TGF‐β family cytokines, including myostatin, nor of type I or type II activin receptors [33]. Taken together, these findings raise questions about the relevance of activin receptor signaling in the pathogenesis of muscle wasting in cancer cachexia.

In conclusion, these data provide proof‐of‐principle that mechanistically distinct strategies targeting muscle anabolism and appetite can act additively with GDF‐15 neutralization, particularly in cancer cachexia contexts characterized by lower dependence on GDF‐15. Future mechanistic validation efforts should prioritize human‐centric approaches, leveraging recent advances in omics technologies. For example, new human muscle transcriptomic data addressing the disease state offers direct insight into disease‐relevant biology [33]. In parallel, large‐scale human genetic studies now enable the disentanglement of muscle and adipose biology from BMI, a historically confounded measure, thereby facilitating genetic validation of muscle‐relevant pathways [34]. In addition, the expanding availability of plasma proteomic datasets, encompassing both large population cohorts (e.g., UK Biobank [35]) and deeply phenotyped studies (e.g., TRACERx [15]), provides an opportunity to interrogate systemic correlates of cachexia. Collectively, a strategy that integrates convergent human genetic, transcriptomic and proteomic evidence to identify mechanisms linked to both muscle mass and function would offer compelling, translatable mechanistic hypotheses.

## Funding

This study was sponsored by Pfizer.

## Ethics Statement

This study involved human participants. The protocol, protocol amendments, informed consent form, Investigator Brochure and other relevant documents (e.g., advertisements) were submitted to the appropriate Institutional Review Board (IRB) or Independent Ethics Committee (IEC) by the investigator and reviewed and approved by the IRB/IEC before the study was initiated. This study was conducted in accordance with the protocol and consensus ethical principles derived from international guidelines, including the Declaration of Helsinki Council and CIOMS International Ethical Guidelines, applicable ICH GCP Guidelines, applicable ISO 14155 guidelines, medical device guidelines and other applicable laws and regulations, including privacy laws. Participants or their legally authorized representatives were informed that their participation was voluntary. Participants or their legally authorized representatives signed a statement of informed consent before enrollment in the study.

## Consent

The authors have nothing to report.

## Conflicts of Interest

B.B., D.B., D.M.B., J.C.S., L.L., M.I.R., R.M.E. and S.C. are employees of Pfizer and may own stock and/or stock options. B.B.Z., B.L.P., J.Y.K‐M., M.P., S.J., S.Q. and Z.W. were employees of Pfizer at the time of study conduct and may own stock.

## Supporting information


**Table S1:** Terminal tumour weights by treatment group in each tumour model study.
**Table S2:** Skeletal muscle tissue weights (mg) by treatment group across mechanistic models.
**Table S3:** Summary of circulating cachexia biomarker concentrations (ng/mL) in each tumour model.
**Figure S1:** Anti‐GDF‐15 improves cachexia in the HT‐1080 tumour model, but not in the presence of caloric restriction. Treatment with anti‐GDF‐15 antibody improved (a) body weight, and increased (b) tumour size, (c) lean mass and (d) fat mass, but not in the presence of caloric restriction (a–d) (*n* = 10–11 per group). NTB and HT‐1080 groups received the vehicle, IgG. Data are mean ± SEM. ****p < 0.0001 vs. NTB; ^#^ *p* < 0.05, ^####^ *p* < 0.0001 vs. HT‐1080; ^^^^*p* < 0.0001 vs. HT‐1080 + Anti‐GDF‐15. ANA, anamorelin; GDF‐15, growth differentiation factor‐15; IgG, immunoglobulin G; SEM, standard error of the mean.
**Figure S2:** Ghrelin receptor agonism‐induced cumulative food intake is attenuated by rhGDF‐15 in healthy mice (*n* = 10 per group). Mice received vehicle or the ghrelin receptor agonist, anamorelin, in the presence or absence of rhGDF‐15. Data are mean ± SEM. ****p < 0.0001 vs. Vehicle + Vehicle; ^#^ *p* < 0.05 vs. Anamorelin + Vehicle. rhGDF‐15, recombinant human growth differentiation factor‐15; SEM, standard error of the mean.
**Figure S3:** In clinical trial participants with advanced NSCLC, circulating plasma levels of (a) activin A and (b) GDF‐11 are not associated with weight loss. Data are shown as geometric mean and 95% confidence intervals by weight loss groups (*n* = 63 for > 5%; *n* = 58 for 0%–5%; *n* = 43 for Stable/Gain [participants who demonstrated stable weight or weight gain]) (ARCHER1009 study; Ramalingam SS et al. *Lancet Oncol* 2014;15:1369–1378). Exogenous administration of GDF‐11 (human) in healthy mice is associated with dose‐related increases in plasma concentrations of (c) GDF‐11 (*n* = 7–10 per group) and (d) GDF‐15 (*n* = 5–10 per group), with concomitant dose‐related reductions in body weight (*n* = 10 per group) as (e) absolute values, and (f) percent of Day 0. Mouse data are individual data points for GDF‐11 and GDF‐15 concentrations with mean and SEM provided (c and d). Mean and SEM are shown for body weight (e and f). ***p < 0.001, ****p < 0.0001 vs. Control AAV 1 × 10^12^. AAV, adeno‐associated virus; GDF, growth differentiation factor; NSCLC, non–small‐cell lung cancer; SEM, standard error of the mean.

## Data Availability

Upon request and subject to review, Pfizer will provide the data supporting this study's findings. Subject to specific criteria, conditions and exceptions, Pfizer may also provide access to the related individual de‐identified participant data. See https://www.pfizer.com/science/clinical‐trials/trial‐data‐and‐results for more information.
