# Supplementary material for: Targeting Cancer Cachexia: A Mechanistic Evaluation of Anti‐GDF‐15 Antibody‐Based Combination Therapies
Source: J Cachexia Sarcopenia Muscle. 2026 May 13;17(3):e70312. doi: 10.1002/jcsm.70312 (PMC13170096; doi:10.1002/jcsm.70312)

**SUPPLEMENTARY MATERIALS**

**Targeting cancer cachexia: A mechanistic evaluation of anti-GDF-15 antibody-based combination therapies**

**Authors:** Danna M. Breen^1*^, Stephanie Joaquim^1^, Brianna LaCarubba Paulhus^1^, Donald Bennett^2^, Barbara Bernardo^1^, Susie Collins^3^, Ryan M. Esquejo^1^, Ja Young Kim-Muller^1^, Laura Lin^4^, Matthew Peloquin^1^, Zhidan Wu^1^, Shuxi Qiao^1^, John C. Stansfield^2^, Bei B. Zhang^1^, Michelle I. Rossulek^1^

***Affiliation:*** *^1^Internal Medicine Research Unit, Pfizer Inc, 1 Portland Street, Cambridge, MA, USA; ^2^Biostatistics, Early Clinical Development, Pfizer Inc, 1 Portland Street, Cambridge, MA, USA; ^3^Statistics, Internal Medicine Research Unit, Pfizer R&D UK Limited, Cambridge, UK; ^4^Biomedicine Design, Pfizer Inc, 1 Portland Street, Cambridge, MA, USA*

***Corresponding author**: Danna M. Breen, Pfizer Inc, 1 Portland Street, Cambridge, MA, USA. Email: [danna.breen@pfizer.com](mailto:danna.breen@pfizer.com); Phone: (617) 551-3000

**METHODS**

**Experimental Models and Subject Details**

***Animals***

Female severe combined immunodeficient CB17 ICR-Prkdc mice (8–10 weeks old) were obtained from Charles River Laboratory (Stone Ridge, NY, USA) and used for the HT-1080 (human fibrosarcoma cell line) and TOV21G (human ovarian cancer cell line) tumor model experiments. Female BALB/c #BALB - Balb/cAnNTac (8–10 weeks old) were obtained from Taconic Biosciences (Germantown, NY, USA) and used for the RENCA (murine renal carcinoma cell line) tumor model experiments. Male C57Bl6/J (8–10 weeks old, Fig S2; 18–20 weeks old, Fig 6) were obtained from Jackson Laboratory (Farmington, CT, USA). All mice were maintained on a 12-h:12-h light-dark cycle and were allowed ad libitum access to water and food (Purina^®^ rodent diet 2029; Purina Mills, St. Louis, MO, USA). All mice were housed individually at thermoneutral conditions (~27°C) (except for Fig 6 and Fig S2, where standard housing temperature (~20°C) was used). Prior to tumor implantation, mice were acclimated to experimental conditions for 2 weeks. The tumor implantation was conducted when the animals were ~10 weeks old, when the animals reached a stable stage in growth and weight gain based on the growth chart from Charles River Laboratory; the length of the studies was 7–8 weeks.

***HT-1080 cell culture***

HT‐1080 cells (HT‐1080 [HT1080] [ATCC^®^ CCL‐121™]) were purchased from the American Type Culture Collection (ATCC; Manassas, VA, USA). Cells were maintained in Eagle’s minimum essential medium supplemented with 10% (v/v) heat‐inactivated fetal bovine serum (FBS), 1% minimum non‐essential amino acids (NEAA), 1% GlutaMAX™ (Thermo Fisher Scientific; Waltham, MA, USA), and 1% penicillin/streptomycin (GIBCO^®^; Thermo Fisher Scientific). The cells were sub‐cultured until the desired number of cells was reached to be used in vivo. At the time of the implant, cells were harvested, counted, and re-suspended into 1:1 PBS.

***RENCA cell culture***

RENCA cells (ATCC CRL-2947™) were purchased from ATCC. Cells were maintained in culture per vendor recommendations; the base medium for this cell line is ATCC-formulated RPMI-1640 medium with 10% FBS, NEAA (0.1mM), sodium pyruvate (1mM), and L-glutamine (2mM). The cells were sub‐cultured until the desired number of cells was reached to be used in vivo. At the time of the implant, cells were harvested, counted, and re-suspended into 1:1 sterile PBS.

***TOV21G cell culture***

TOV21G cells (TOV21G [ATCC CCL-11730 lot 63990061]) were purchased from ATCC. Cells were maintained in a 1:1 mixture of MCDB 105 medium containing a final concentration of 1.5 g/L sodium bicarbonate and medium 199 containing a final concentration of 2.2 g/L sodium bicarbonate and supplemented with 15% FBS and 1% penicillin/streptomycin (Thermo Fisher Scientific). The cells were sub‐cultured until the desired number of cells was reached to be used in vivo. At the time of the implant, cells were harvested, counted, and re-suspended into 1:1 sterile PBS.

**Generation of the Tumor Models and Tumor Growth Measurements**

***HT-1080***

Five million cells per animal were implanted subcutaneously in a 200-mL injection of cell suspension (5 million cells suspended in equal parts of sterile Matrigel^®^ [Corning, Tewksbury, MA, USA] and PBS) in the flank region without anesthesia. Tumor growth was monitored daily following inoculation and size was measured every 2–3 days using digital calipers (Mitutoyo, 500-171-30) when a palpable tumor was formed. Tumor weight was estimated using the following equation: Tumor weight = (width)^2^ x length / 2.

***RENCA***

Ten million cells per animal were implanted subcutaneously in a 200-mL injection of cell suspension (10 million cells suspended in equal parts of sterile Matrigel and PBS) in the flank region without anesthesia. Tumor growth was monitored daily following inoculation and size was measured every 2–3 days using digital calipers (Mitutoyo, 500-171-30) when a palpable tumor was formed. Tumor weight was estimated using the following equation: Tumor weight = (width)^2^ x length / 2.

***TOV21G***

Five million cells per animal were implanted subcutaneously in a 100-mL injection of cell suspension (5 million cells suspended in equal parts of sterile Matrigel and PBS) in the flank region without anesthesia. Tumor growth was monitored daily following inoculation and size was measured once per week using digital calipers (Mitutoyo 500-171-30) when a palpable tumor was formed. Tumor weight was estimated using the following equation: Tumor weight = (width)2 x length / 2.

**Quantification and Statistical Analysis**

Statistical analyses were conducted using R (version 4.0.5). Longitudinal mixed-effects models with autoregressive (1) covariance structures, fixed effects for treatment group and time, and random intercepts for each animal were used to compare longitudinal body weight measurements, tumor mass, and wheel running over specified time intervals. For in vivo muscle function measurements, a repeated measures mixed-effects model with an autoregressive (1) covariance structure, with fixed effects for treatment group and frequency and a random intercept for each mouse was used to compare muscle function at 125 Hz and 150 Hz. One-way analysis of variance (ANOVA) and Tukey’s Honestly Significant Difference tests were used for comparing all continuous endpoints between multiple treatments at a single time point. Circulating mouse and human GDF-15 levels in non–tumor-bearing and TOV21G tumor-bearing mice were compared by a Mann-Whitney U test. For the clinical samples, statistical analyses were conducted using SAS (SAS Institute, Cary, NC, USA). Patients were classified into weight-loss groups. Each plasma variable was analyzed on the log scale using ANOVA, followed by pairwise comparisons to the stable/gain weight-loss group. Data were back-transformed for presentation as geometric means and 95% confidence intervals.

**RESULTS**

**Table S1.** Terminal tumor weights by treatment group in each tumor model study.

| **Tumor Model** | **Treatment Groups** | **Terminal Tumor Weight (g), mean (± SEM)** |
| --- | --- | --- |
| HT-1080 (Study 1) | HT-1080 | 0.57 (0.09) |
|  | HT-1080+Anti-myo | 0.91 (0.12) |
|  | HT-1080+Anti-GDF-15 | 0.91 (0.30) |
| HT-1080 (Study 2) | HT-1080 | 0.94 (0.11) |
|  | HT-1080+Anti-GDF-15 | 1.96 (0.24)^##^ |
|  | HT-1080+Anti-GDF-15+Anti-myo | 1.85 (0.18)^##^ |
| RENCA | RENCA | 0.54 (0.05) |
|  | RENCA+Anti-myo | 0.48 (0.06) |
|  | RENCA+Anti-GDF-15 | 0.52 (0.05) |
|  | RENCA+Anti-myo+Anti-GDF-15 | 0.50 (0.08) |
| TOV21G | TOV21G | 0.58 (0.03) |
|  | TOV21G+Anti-GDF-15 | 0.67 (0.05) |
|  | TOV21G+Anti-myo+Anti-GDF-15 | 0.66 (0.05) |
| HT-1080 (Ghrelin) | HT-1080 | 1.28 (0.23) |
|  | HT-1080+ANA | 0.94 (0.20) |
|  | HT-1080+Anti-GDF-15 | 1.08 (0.17) |
|  | HT-1080+ANA+Anti-GDF-15 | 1.04 (0.15) |

^##^*p*<0.01 vs. HT-1080. Vehicle = IgG Control.

ANA, anamorelin; Anti-myo, anti-myostatin; GDF-15, growth differentiation factor-15; IgG, immunoglobulin G; SEM, standard error of the mean.

**Table S2.** Skeletal muscle tissue weights (mg) by treatment group across mechanistic models.

| **Model** | **Treatment Groups** | **Gastrocnemius** | **Tibialis anterior** | **Quadriceps** |
| --- | --- | --- | --- | --- |
| HT-1080 | NTB | ^a^146 (5) | ^b^45 (2) | 199 (3) |
| (**Fig 2e**) | NTB+Anti-GDF-15 | ^a^147 (4) | ^b^43 (1) | 194 (6) |
|  | NTB+Anti-GDF-15+Anti-myo | ^a^156 (4) | ^b^48 (2) | 209 (5) |
|  | HT-1080 | ^a^114 (4)**** | ^b^37 (2)* | 161 (6)*** |
|  | HT-1080+Anti-GDF-15 | ^a^135 (4)^##^ | ^b^39 (1) | 184 (6)^#^ |
|  | HT-1080+Anti-GDF-15+Anti-myo | ^a^147 (3)^####^ | ^b^43 (2)^#^ | 192 (6)^##^ |
| RENCA | NTB | ^a^157 (10) | 32 (1) | 117 (7) |
| (**Fig 3e**) | RENCA | ^a^107 (5)** | 29 (1) | 93 (4)* |
|  | RENCA+Anti-myo | ^a^131 (9) | 30 (1) | 106 (5) |
|  | RENCA+Anti-GDF-15 | ^a^141 (10)^#^ | 32 (1) | 121 (3)^###^ |
|  | RENCA+Anti-myo+Anti-GDF-15 | ^a^162 (8)^####^ | 36 (1)^####^ | 121 (6)^###^ |
| TOV21G | NTB | ^a^135 (4) | 36 (2) | 169 (6) |
| (**Fig 4f**) | TOV21G | ^a^104 (8)**** | 30 (2)** | 130 (7)**** |
|  | TOV21G+Anti-GDF-15 | ^a^131 (5)^####^ | 34 (2)^#^ | 165 (6)^####^ |
|  | TOV21G+Anti-GDF-15+Anti-myo | ^a^145 (5)^####^ | 42 (4)^####,$$$$^ | 195 (7)^####,$$$$^ |
| Fc-GDF-15 | IgG | ^a^174 (7) | ^b^58 (5) | 218 (10) |
| (**Fig 6c**) | Anti-myo | ^a^199 (6)*** | ^b^66 (4)** | 251 (10)** |
| Fc-GDF-15 | Vehicle | 181 (4) | 52 (1) | 234 (7) |
| **(Fig 6f)** | Fc-GDF-15 | 156 (3)**** | 45 (2)** | 206 (4)* |
|  | Fc-GDF-15+ Anti-myo | 166 (3) | 50 (1) | 226 (4)^#^ |
|  | Fc-GDF-15+ Anti-GDF-15 | 179 (3)^###^ | 52 (2)^##^ | 228 (4)^#^ |

All data are expressed as mean (SEM). NTB and tumor groups received the vehicle, IgG.

^a^Weight includes the gastrocnemius and soleus combined.

^b^Weight includes the tibialis anterior and extensor digitorum longus combined.

****P<0.0001, ***P<0.001, **P<0.01, *P<0.05 vs NTB or Veh.

^####^P<0.0001, ^###^P<0.001, ^##^P<0.01, ^#^P<0.05 vs Tumor or Fc-GDF-15.

^$$$$^P<0.0001 vs Tumor+Anti-GDF-15.

Anti-myo, anti-myostatin; Fc-GDF-15, GDF-15 optimized in an Fc-fusion protein format for improved half-life; GDF-15, growth differentiation factor-15; IgG, immunoglobulin G; NTB, non-tumor bearing; SEM, standard error of the mean.

**Table S3.** Summary of circulating cachexia biomarker concentrations (ng/ml) in each tumor model.

| **Tumor Type** | **GDF-15** | **Myostatin** | **Activin A** | **GDF-11** |
| --- | --- | --- | --- | --- |
| HT-1080 | 4.2 ± 0.6 | 35 ± 3 | 0.3 ± 0.05^†^ | 0.9 ± 0.05^†^ |
| RENCA | 2.9 ± 0.9 | 67 ± 4 | 0.3 ± 0.05 | ND |
| TOV21G | 0.9 ± 0.3 | 59 ± 9 | 0.9 ± 0.03^‡^ | ND |

All data are expressed as mean (SEM).

^†^Bernardo B, et al. *J Cachexia Sarcopenia Muscle* 2020;11:1813-1829.

^‡^Lerner L, et al. *J Cachexia Sarcopenia Muscle* 2016;7:467-482.

GDF, growth differentiation factor; ND, not determined; SEM, standard error of the mean.

**Fig S1**. Anti-GDF-15 improves cachexia in the HT-1080 tumor model, but not in the presence of caloric restriction. Treatment with anti-GDF-15 antibody improved (**a**) body weight, and increased (**b**) tumor size, (**c**) lean mass, and (**d**) fat mass, but not in the presence of caloric restriction (**a–d**) (*n*=10–11 per group). NTB and HT-1080 groups received the vehicle, IgG.

Data are mean ± SEM.

*****p*<0.0001 vs. NTB; ^#^*p*<0.05, ^####^*p*<0.0001 vs. HT-1080; ^^^^*p*<0.0001 vs. HT-1080 + Anti-GDF-15.

ANA, anamorelin; GDF-15, growth differentiation factor-15; IgG, immunoglobulin G; SEM, standard error of the mean.


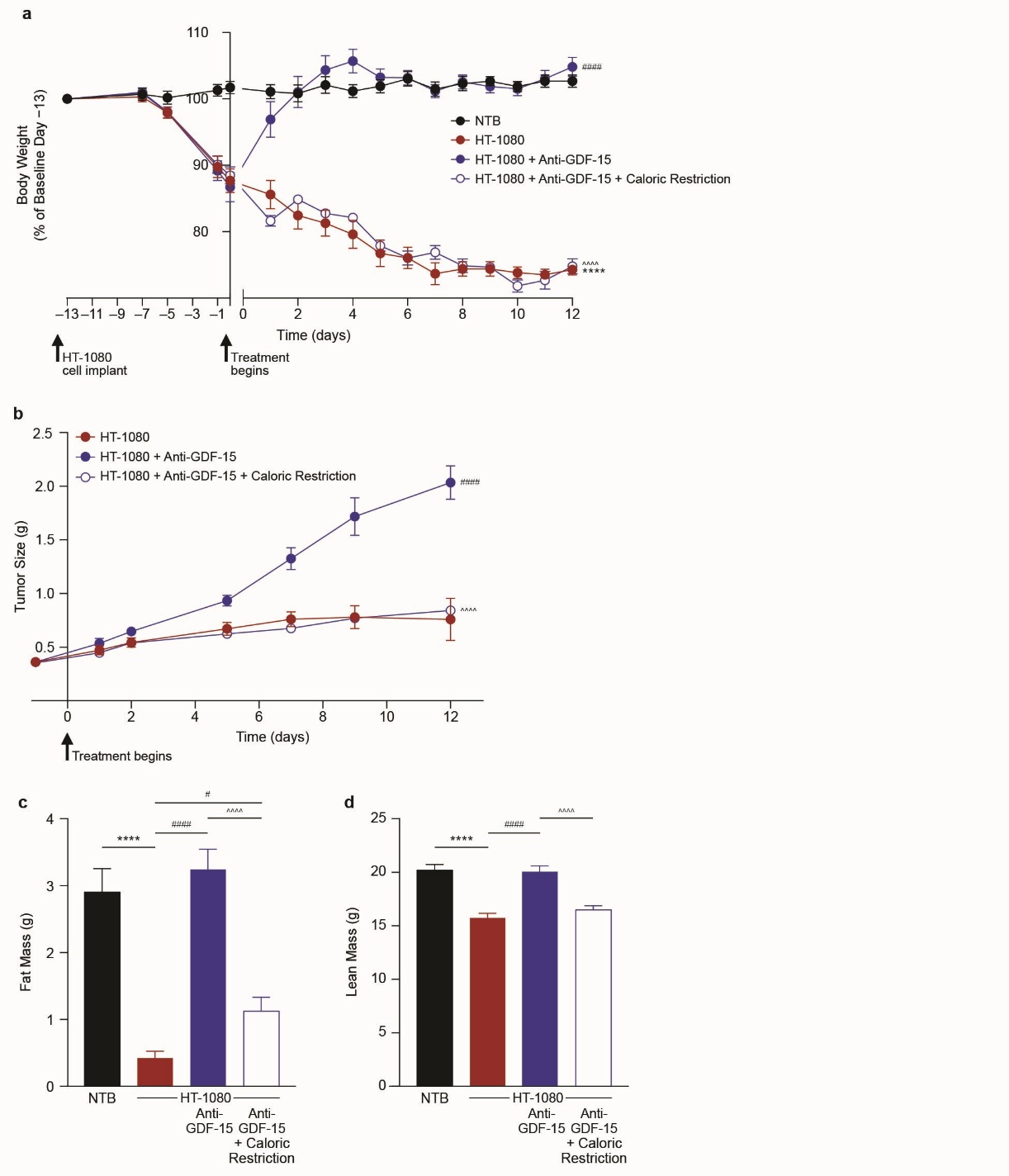


**Fig S2.** Ghrelin receptor agonism-induced cumulative food intake is attenuated by rhGDF-15 in healthy mice (*n*=10 per group). Mice received vehicle or the ghrelin receptor agonist, anamorelin, in the presence or absence of rhGDF-15.

Data are mean ± SEM.

*****p*<0.0001 vs. Vehicle + Vehicle; ^#^*p*<0.05 vs. Anamorelin + Vehicle.

rhGDF-15, recombinant human growth differentiation factor-15; SEM, standard error of the mean.


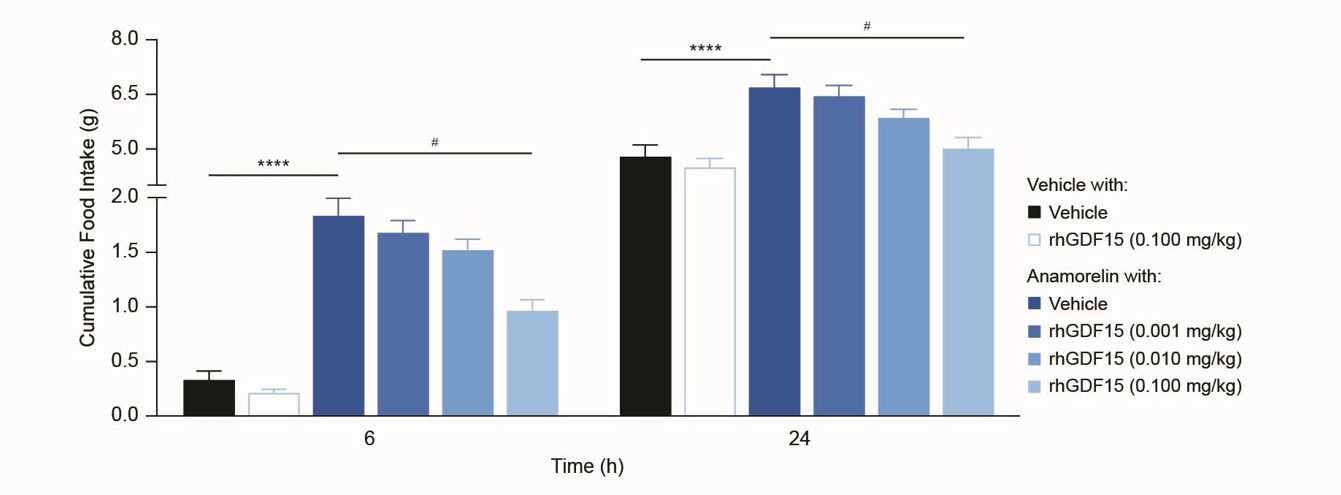


**Fig S3**. In clinical trial participants with advanced NSCLC, circulating plasma levels of (**a**) activin A and (**b**) GDF-11 are not associated with weight loss. Data are shown as geometric mean and 95% confidence intervals by weight loss groups (n=63 for >5%; n=58 for 0-5%; n=43 for Stable/Gain [participants who demonstrated stable weight or weight gain]) (ARCHER1009 study; Ramalingam SS, et al. *Lancet Oncol* 2014;15:1369-1378). Exogenous administration of GDF-11 (human) in healthy mice is associated with dose-related increases in plasma concentrations of (**c**) GDF-11 (*n*=7–10 per group) and (**d**) GDF-15 (*n*=5–10 per group), with concomitant dose-related reductions in body weight (*n*=10 per group) as (**e**) absolute values, and (**f**) percent of Day 0. Mouse data are individual data points for GDF-11 and GDF-15 concentrations with mean and SEM provided (**c** and **d**). Mean and SEM are shown for body weight (**e** and **f**).

***p<0.001, ****p<0.0001 vs. Control AAV 1x10^12^.

AAV, adeno-associated virus; GDF, growth differentiation factor; NSCLC, non–small-cell lung cancer; SEM, standard error of the mean.


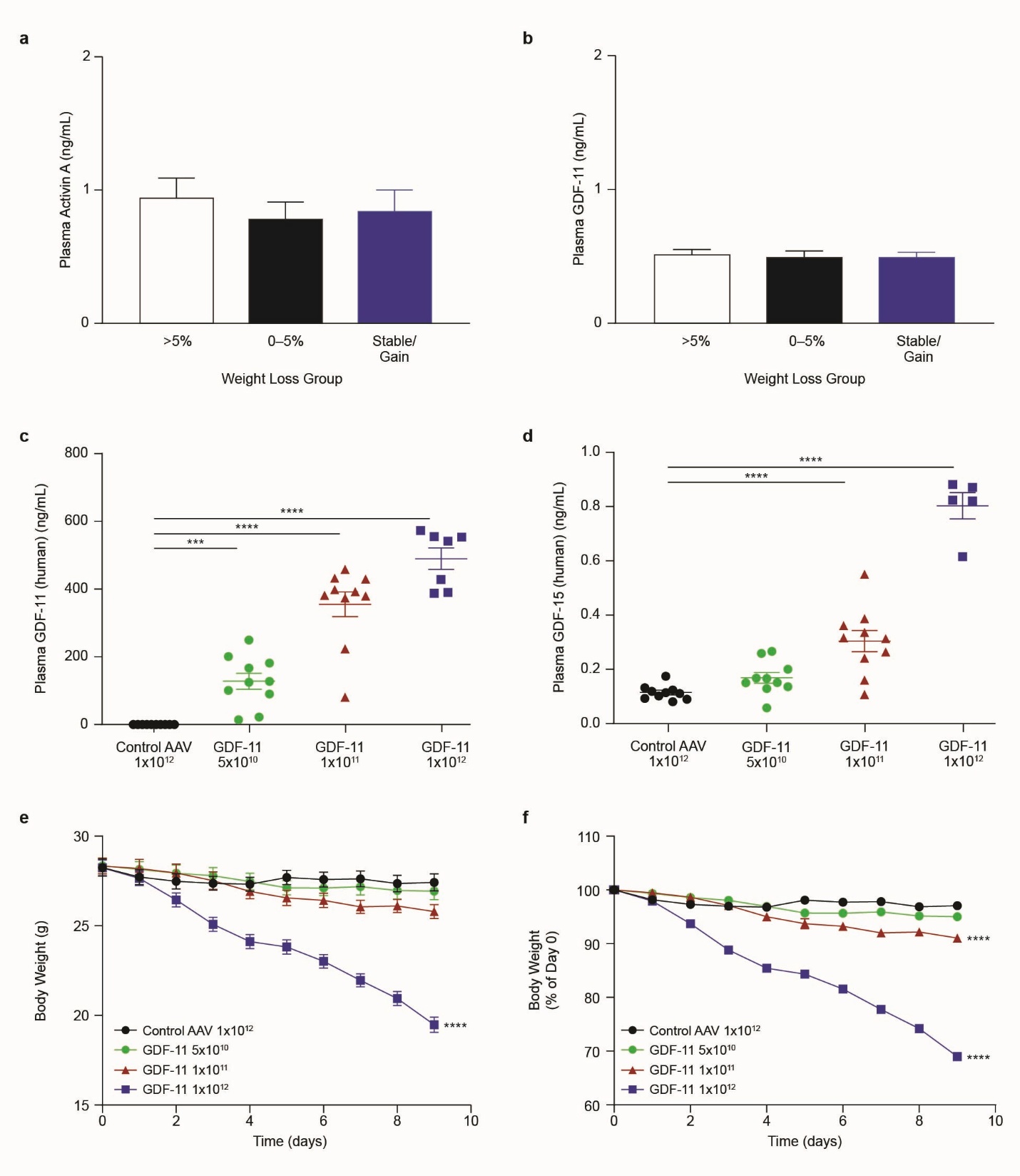


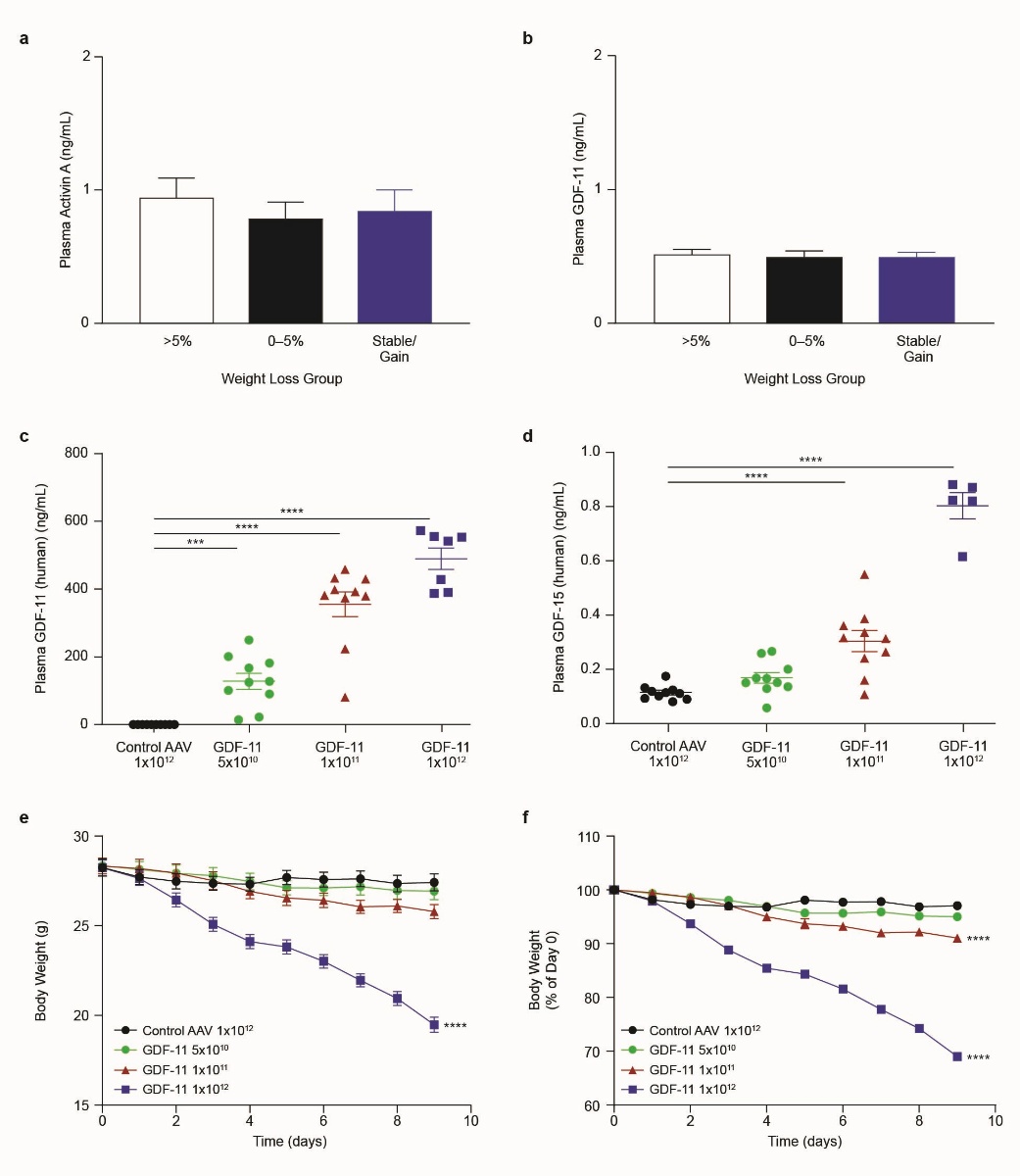

Supplement: Supplementary file 1 — Table S1: Terminal tumour weights by treatment group in each tumour model study. Table S2: Skeletal muscle tissue weights (mg) by treatment group across mechanistic models. Table S3: Summary of circulating cachexia biomarker concentrations (ng/mL) in each tumour model. Figure S1: Anti‐GDF‐15 improves cachexia in the HT‐1080 tumour model, but not in the presence of caloric restriction. Treatment with anti‐GDF‐15 antibody improved (a) body weight, and increased (b) tumour size, (c) lean mass and (d) fat mass, but not in the presence of caloric restriction (a–d) (n = 10–11 per group). NTB and HT‐1080 groups received the vehicle, IgG. Data are mean ± SEM. ****p < 0.0001 vs. NTB; # p < 0.05, #### p < 0.0001 vs. HT‐1080; ^^^^p < 0.0001 vs. HT‐1080 + Anti‐GDF‐15. ANA, anamorelin; GDF‐15, growth differentiation factor‐15; IgG, immunoglobulin G; SEM, standard error of the mean. Figure S2: Ghrelin receptor agonism‐induced cumulative food intake is attenuated by rhGDF‐15 in healthy mice (n = 10 per group). Mice received vehicle or the ghrelin receptor agonist, anamorelin, in the presence or absence of rhGDF‐15. Data are mean ± SEM. ****p < 0.0001 vs. Vehicle + Vehicle; # p < 0.05 vs. Anamorelin + Vehicle. rhGDF‐15, recombinant human growth differentiation factor‐15; SEM, standard error of the mean. Figure S3: In clinical trial participants with advanced NSCLC, circulating plasma levels of (a) activin A and (b) GDF‐11 are not associated with weight loss. Data are shown as geometric mean and 95% confidence intervals by weight loss groups (n = 63 for > 5%; n = 58 for 0%–5%; n = 43 for Stable/Gain [participants who demonstrated stable weight or weight gain]) (ARCHER1009 study; Ramalingam SS et al. Lancet Oncol 2014;15:1369–1378). Exogenous administration of GDF‐11 (human) in healthy mice is associated with dose‐related increases in plasma concentrations of (c) GDF‐11 (n = 7–10 per group) and (d) GDF‐15 (n = 5–10 per group), with concomitant dose‐related reductions in body weight ( [file JCSM-17-e70312-s001.docx]
